# Factors influencing uptake of diagnostic test interventions for SARS-CoV-2: A qualitative review

**DOI:** 10.4102/jphia.v16i2.619

**Published:** 2025-04-28

**Authors:** Nuria S. Nwachuku, Dachi I. Arikpo, Ugo J. Agbor, Peter N. Onyenemerem, Eleanor A. Ochodo, Helen Smith, Martin Meremikwu

**Affiliations:** 1 Department of Public Health, Faculty of Allied Medical Sciences, University of Calabar, Calabar, Nigeria; 2 Cochrane Nigeria, Institute of Tropical Diseases Research and Prevention, University of Calabar Teaching Hospital, Calabar, Nigeria; 3 Cross River Health and Demographic Surveillance System, University of Calabar, Calabar, Nigeria; 4 Johns Hopkins Program for International Education in Gynecology and Obstetrics (Jhpiego), Uyo, Nigeria; 5 Centre for Global Health Research, Kenya Medical Research Institute, Nairobi, Kenya; 6 Department of Global Health, Faculty of Medicine and Health Sciences, Stellenbosch University, Cape Town, South Africa; 7 International Health Consulting Services Ltd, Merseyside, United Kingdom; 8 Department of Pediatrics, University of Calabar, Calabar, Nigeria

**Keywords:** infection prevention, diagnostic test, screening, asymptomatic, COVID-19

## Abstract

**Background:**

Diagnostic tests for severe acute respiratory syndrome coronavirus-2 (SARS-CoV-2) may be performed based on symptomatic presentation or for screening of asymptomatic persons. Testing can limit spread by enabling rapid identification of cases leading to containment measures. However, views regarding diagnostic test as a containment intervention vary across different settings.

**Aim:**

To synthesise the findings of qualitative studies on the perceptions and factors influencing the uptake of diagnostic test interventions for SARS-CoV-2.

**Setting:**

Healthcare facilities, care homes, communities including households.

**Method:**

We searched MEDLINE database and the (WHO) SARS-CoV-2 Research database from 01 January 2020 to 06 September 2022. Qualitative data were synthesised thematically while data for barriers and facilitators were synthesised using the SURE framework. The GRADE-CERQual approach was used to assess the confidence in each review finding, while the ENTREQ checklist was used to report the QES. The quality of included studies was assessed with the CASP tool.

**Results:**

Twenty two studies were included for QES. Two were conducted in the health facility setting, 2 in care homes, and 18 in the community. Twenty of the studies came from high-income countries, 2 from low- and middle-income countries. In all, 13 analytical and 31 descriptive themes of low to moderate quality evidence emerged; mainly around fear of contracting COVID-19, test procedure and socio-economic implications of a positive test result.

**Conclusion:**

Low to moderate quality evidence of barriers to uptake of diagnostic test were largely due to misconceptions about the interventions.

**Contribution:**

Sensitising and engaging communities and stakeholders in the healthcare system, will help mitigate the fear barrier and enhance policy coordination.

## Introduction

A variety of infection prevention and control (IPC) strategies were adopted in response to the global severe acute respiratory syndrome coronavirus 2 (SARS-CoV-2) pandemic, including use of personal protective equipment (PPE), face masks, physical distancing, proper hand hygiene, respiratory etiquette, cleaning and disinfection, proper ventilation as well as early identification of cases through testing and subsequent isolation.^1,2,3,4,5^ Diagnostic tests for coronavirus disease 2019 (COVID-19) help to limit spread and enable rapid identification of cases so that appropriate containment measures can be implemented, such as isolation.^6,7,8,9,10^ Furthermore, testing is essential in assessing epidemiological situations globally and is also required to drive the controlled resumption of social and economic activities globally.^11,12,13^

Affordability, availability and access to these diagnostic tests could pose a challenge especially in resource constrained settings and will likely influence uptake of these interventions aimed at containing the spread of the disease.^14,15,16^ Successfully preventing or managing outbreaks through diagnostic tests depends on multiple factors that may act as barriers or facilitators to uptake; these factors cut across the individual, family, community as well as at the organisational levels.^17,18,19,20^ Availability, acceptability, accessibility, affordability are key issues that drive the success of the diagnostic test strategy.^21,22,23,24^

Different stakeholders and clients may have varying views and perceptions of diagnostic tests, based on the context in which they live and work.^25,26^ Furthermore, in some settings where these tests are available, accessible and affordable, uptake have been less than adequate prompting questions on barriers to uptake.^23^ Therefore, it is important to have an in-depth understanding of contextual factors through qualitative research that may hinder uptake of diagnostic tests across different settings, especially with the advent of rapid diagnostic tests (RDTs), which are easy to operate and available right at the point of care. Identifying positive individuals through diagnostic tests will help reduce the risk of severe illness and risk of long-term disability or death for those infected and reduce the spread of the virus.^10^ To the best of our knowledge, this is the first qualitative systematic review on perceptions and factors influencing uptake of diagnostic test interventions for IPC in the context of COVID-19.

### Aim

The aim of this review is to identify and synthesise the findings of qualitative studies on the perceptions, experiences and views of healthcare providers, recipients of care and community members on diagnostic test interventions and on barriers and facilitators to uptake of diagnostic test interventions in the context of COVID-19. This synthesis was conducted as part of a routine update of the World Health Organization (WHO) guidelines on IPC in the context of COVID-19.

## Methods

### Design

We conducted a systematic review of qualitative studies following the methods described in the Cochrane handbook of systematic reviews and outlined in the Cochrane guidance on conducting rapid qualitative evidence synthesis (QES).^27,28,29^ The review protocol was registered and published with the International Prospective Register of Systematic Reviews (PROSPERO, CRD42022356698). Findings from this rapid QES are reported using the enhancing transparency in reporting the synthesis of qualitative research (ENTREQ) checklist.^30^

### Search strategy

We searched MEDLINE (Ovid) and the WHO COVID-19 register from 01 January 2020 to 07 September 2022. We also searched the reference list of all included studies, including related systematic reviews, to identify any additional potentially eligible studies for inclusion. We used terms such as ‘COVID-19 Testing or COVID*’ or ‘SARS-CoV-2’ or ‘coronavirus*’ or ‘COV’ or ‘NCOV’ for Medline, and for WHO COVID-19 register, we used ‘(test or tests or testing) AND (transmission or replication or prevent* or transmit* or spread* or contain or containment or proliferat*) and (‘adhere to’ or adherence or attitude* or barriers or behaviour or behaviour or challeng* or compliance or comply* or facilitat* or influenc* or knowledge or perception* or practice*) and (focus group* or qualitative or ethnograph* or fieldwork or ‘field work’ or ‘key informant’ or interview* or discussion* or questionnaire* or survey* or experience* or narration or ‘personal narrative’ or ‘self report’ or type_of_study:(‘qualitative_research’)) and la:(‘en’) and type:(‘article’).

Details of the search strategy, including the search terms, and the Boolean operators for each database are outlined in Online Appendix 1.

### Study selection and sampling

**FIGURE 1:**
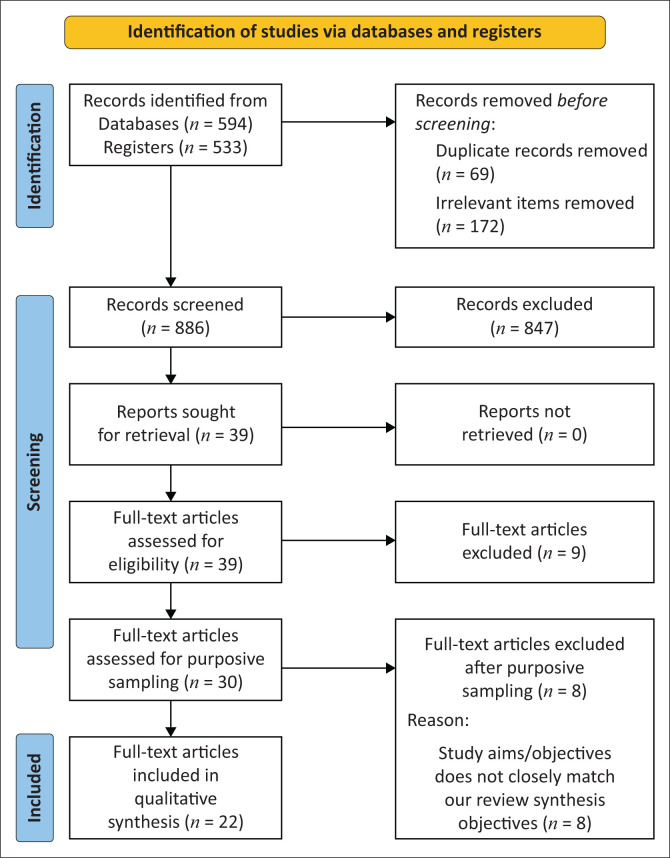
Preferred Reporting Items for Systematic Reviews and Meta-Analyses (PRISMA) flow diagram of included studies.

All search hits were imported into the Endnote Reference Management software where duplicates and irrelevant items were automatically removed. The authors then proceeded with screening of the remaining records in three stages. Titles and abstracts were screened first using an eligibility criteria form, followed by full-text screening and then sampling. Screening was completed in pairs, one author screened all titles, abstracts and full texts of potentially eligible studies using a pre-piloted eligibility screening form. A second author, verified all output from each of these stages. We used the Preferred Reporting Items for Systematic Reviews and Meta-Analyses (PRISMA) guideline and flow diagram to report the search and selection of studies (Figure 1).^31^

Author pairs resolved discrepancies in the study selection process by consulting a third review author. Full-text screening yielded 30 studies. We considered this number too large to analyse adequately, considering the short time frame for the review and therefore purposively sampled papers using maximum variation sampling.^32,33,34^ We developed a three-step sampling frame with the following parameters: closeness of the study to our synthesis objective, geographical spread or representation and data richness using the data richness scale.^35^

Online Appendix 2 provides references and details of included but not sampled studies.

### Inclusion criteria

Table 1 outlines the inclusion criteria. We used the setting, perspective, phenomenon of interest, comparison and evaluation (SPICE) framework to help shape the review question and articulate the inclusion criteria.^36^ Because of the short time frame of the review, we included only published studies in English. We did not exclude any study based on our assessment of methodological limitations.

**TABLE 1:** Inclusion criteria.

SPICE	Eligibility framework
Setting	Healthcare facilities, including care homes Community including households In any geographical location and level of healthcare
Perspective (population)	Stakeholders: Healthcare workers involved in patient care Healthcare personnel not involved in patient care Health care policy makers Health facility clients (including residents of care homes, inpatients, outpatients) and visitors Community members and public members of households
Phenomenon of interest	Diagnostic test interventions for COVID-19 infection prevention and control
Intervention	All types of diagnostic tests used for *in vitro* diagnosis of both symptomatic and asymptomatic persons, for example: Nucleic acid amplification test (NAAT)-Reverse transcription polymerase chain reaction (RT-PCR) Serological tests-Antigen and Antibody based test including rapid diagnostic tests (RDTs) Enzyme-linked immunosorbent assays (ELISAs) Chemiluminescent immunoassays (CLIAs)
Evaluation (outcome)	Perceptions of stakeholders, including views, attitudes, experiences and perspectives Factors influencing uptake (barriers and facilitators) at the individual, provider, health system, community and social-political levels
Study design	Primary studies conducted using qualitative study designs, including ethnography, phenomenology, case studies, grounded theory studies, applied qualitative research, mixed methods and process evaluations Studies using qualitative methods for data collection (e.g., focus group discussions, individual interviews, observation) and qualitative methods for data analysis (e.g., thematic analysis, framework analysis, content analysis and grounded theory)
Date limits	01 January 2020 to 07 September, 2022; to capture research published in response to the COVID-19 outbreak.

### Data extraction

Key study characteristics and outcomes were extracted using a pre-piloted data extraction spreadsheet in Microsoft (MS) Excel. Two additional MS Excel spreadsheet were used to extract themes and supporting quotes relevant to the review objectives. Details of themes and supporting quotes are presented in Table 3. For facilitators and barriers, we extracted information on factors at the individual, provider, health system, community and social-political levels, and mapped this onto elements of the Supporting the use of Research Evidence (SURE) framework.^37^ Two review authors (U.J.A. and P.N.O.) extracted data from the sampled studies, and one author (N.S.N.) verified all extracted data for accuracy and completeness. Disagreements were resolved by a third author (D.I.A.) or by consulting other review authors in the team.

### Assessment of methodological limitations of sampled studies

An adapted version of the Critical Appraisal Skills Programme (CASP) tool for qualitative studies^37^ was used for assessment of the methodological limitations of the sampled studies. The tool adapted, contains seven items evaluating the (1) appropriateness or adequacy of descriptions of the study context and setting(s), (2) sampling strategy, (3) data collection, (4) data analysis, (5) evidence supporting the findings, (6) evidence of reflexivity and (7) ethical considerations. No study was excluded based on the quality assessment.

### Review author reflexivity

All the authors experienced the COVID-19 pandemic and generally considered IPC strategies essential for mitigating the spread of the virus. Before the review commenced, all authors declared no conflict of interest about the study. Throughout the review process, the authors were mindful of their inclinations. They minimised bias in the analysis and interpretation of the review findings by discussing and agreeing on the review findings as a team. The multi-disciplinary nature of the team allowed for rich insights and balanced views on the findings and interpretation of the evidence.

### Data synthesis

The thematic synthesis approach^38^ was manually used to synthesise relevant qualitative data. This allowed us to generate descriptive themes directly from the data and categorise them using inductive and ‘constant comparison’ methods.^39^ This approach is suitable for exploring the perspectives and experiences of stakeholders^40,41^ and involves familiarisation with the data (initial coding), coding the texts (line-by-line coding), developing descriptive themes and generating analytical themes. To synthesise data on factors influencing uptake of diagnostic test interventions, we used a ‘Best-fit’ framework synthesis method.^42^ The ‘Best-fit’ framework synthesis uses deductive methods to fit the findings of qualitative studies into a pre-existing framework. We adopted the SURE framework^37^ as the appropriate framework for this synthesis because it identifies factors that influence the implementation of a policy option at the level of the care recipient, care provider, health service and system constraints, and the social and political context. Once we had identified descriptive themes, we then refined these into analytical themes. This involved going beyond the content of the original studies to address the aspects important to help the guideline development group use this qualitative evidence to inform their decision-making on the recommendations (D.I.A., H.S., N.S.N. and E.A.O. analysed the data.).

### Assessing confidence in the review findings

We used the Grading of Recommendations Assessment, Development, and Evaluation and the Confidence in the Evidence from Reviews of Qualitative Research (GRADE-CERQual) approach^43^ to assess the confidence level (high, moderate, low or very low) in each review finding. This assessment is made across four domains (methodological limitations of included studies, coherence of the review finding, adequacy of the data contributing to a review finding and relevance of the included studies to the review question). Two review authors assessed the confidence of each finding across the four domains, with the overall assessment based on the consensus of all review authors.

### Ethical considerations

This article followed all ethical standards for research without direct contact with human or animal subjects.

## Results

### Search results

The initial title and abstract screening yielded 39 studies likely for inclusion out of the 886 studies from the search output. After full-text screening, 30 studies met inclusion criteria and were further subjected to purposive sampling which yielded 22 studies for the final QES based on relevance, geographical spread and depth of insight. Details of study selection process are captured in the Preferred Reporting Items for Systematic Reviews and Meta-Analyses (PRISMA) flow diagram (Figure 1). Online Appendix 2 provides the references and characteristics of included but not sampled studies.

### Description of included studies

Table 2 summarises the characteristics of sampled studies. Included studies were from high-income countries (HICs: England, Germany, the Netherlands, South Australia, United States [US]) (*n* = 20) and low- and middle-income countries (LMICs: The Philippines and Nepal) (*n* = 2).

**TABLE 2:** Characteristics of included studies and the overall assessment of limitations.

Study ID	Study setting	Aim of study	Study design	Perspective	Participants	Sample size	Data collection method	Context	Method of data analysis	Overall assessment of methodological limitations
Bateman, 2021^44^ US (HIC)	Community	To examine perceptions of COVID-19 related to prevention, coping, and testing of African American residents in under-resourced communities in Alabama.	Primary qualitative	Community members	African American residents in under-resourced communities in Alabama *who* were either members of the respective coalition or a resident of the community recommended by a coalition member.	36	FGDs	Urban	Thematic analysis	Moderate
Garcini, 2022^23^ US (HIC)	Community	To identify barriers and facilitators to diagnostic testing for COVID-19 among underserved Latino communities, in particular those residing in proximity to the US-Mexico border.	Mixed method	Community members	Community health workers (CHW) and Promotors in Texas.	64	FGDs	Urban	Systematic methods outlined by Miles and Huberman (1994),	Minor
Gehlbach, 2022^26^ US (HIC)	Community	To understand how both structural and SDOH shape perceptions of the coronavirus, its spread, and decision-making around COVID-19 testing and vaccination in vulnerable populations.	Primary Qualitative	Community members	Racial-ethnic minority groups, specifically Latinx and Indigenous Latin American immigrants.	53	FGDs	Rural	Template and matrix analysis	Minor
Gierszewski, 2022^45^ Germany (HIC)	Community	To describe the factors that facilitate or hinder the implementation of continuous SARS-CoV-2 testing from the perspective of parents and children and childcare workers (CCWs) involved in the study.	Primary qualitative	Community members	Parents and childcare workers (CCWs) in day care centres.	76	Interview	-	Qualitative content analysis by Kuckartz using MAXQDA 2020	Minor
Knight, 2022^46^ US (HIC)	Community	To understand the facilitators and barriers to COVID-19 testing and vaccine acceptability among homeless-experienced adults to inform strategies to improve the delivery and uptake of COVID-19 testing and vaccination in this population.	Primary qualitative	Community members	Adults with current or past experience of homelessness.	94	Interview	Urban	Content analysis	Minor
Lorenc, 2021^47^ England (HIC)	Community	The study aimed to rapidly explore student, parent/carer and school staff attitudes towards school COVID-19 mitigation measures, views on managing COVID-19 infections in schools and opinions about student groups who may be particularly affected by these measures.	Primary qualitative	Community members	Student, parents and carers and secondary school staff.	52	Interview	Urban	Framework analysis	Moderate
Mathers, 2022^48^ England (HIC)	Community	To examine public perspectives on Lateral flow testing (LFT) for COVID-19 at a time of national population-level screening and increasing rates of COVID-19 vaccination. The research explored reasons for uptake or refusal of testing in different settings; patterns of testing (frequency, who within households is testing); experience of the testing process; perceptions of test accuracy and behavioural intentions post testing.	Primary qualitative	Community members	Any resident aged 18 years or above in Birmingham City Council catchment area.	21	Interview	Urban	Thematic analysis	Minor
Nwaozuru, 2022^49^ US (HIC)	Community	To explore the acceptability and recommendations to promote and scale-up the uptake of COVID-19 ST among black and African Americans.	Primary qualitative	Community members	Black and African Americans.	28	Open-ended questionnaires	Urban	Inductive content analysis	Minor
Robin, 2022^50^ England (HIC)	Community	To identify barriers and facilitators to engaging in mass asymptomatic testing and to generate recommendations for improving uptake of mass asymptomatic testing in future.	Primary qualitative	Community members	Publicly accessible sources of community narratives, including social and online media sites. (Online comments sections from the local online newspaper for Liverpool City, LCC Facebook page, and Twitter).		local narratives from local community media and social media	Urban	Thematic analysis	Minor
DeRoo, 2021^51^ US (HIC)	Health facility	To characterise knowledge, attitudes, and beliefs about COVID-19 testing among black parents.	Primary qualitative	Community members	Parents after telemedicine visits with a children’s health centre.	26	Interview	Urban	Phenomenological approach based on the Health Belief Model	None
Thorneloe, 2022^52^ England (HIC)	Community	To identify the key factors affecting adherence to test, trace, and isolate behaviours using the Theoretical Domains Framework (TDF).	Primary qualitative	Community members	People living in Shefeld who came into close contact with others in work or social settings.	30	FGDs	Urban	Framework analysis	None
Tonkin, 2022^53^ Australia (HIC)	Community	To explore community members’ decisions about having COVID-19 testing in an environment of low prevalence, specifically exploring their decision-making related to symptoms.	Primary qualitative case	Community members	People who experienced any COVID-19-like symptom(s) since the commencement of testing in Adelaide, South Australia.	29	FGDs	Urban	Framework analysis	Minor
Unger, 2021^54^ US (HIC)	Community	To examine the attitudes of school administrators, teachers, parents, and students towards using COVID-19 testing as part of a strategy to reopen schools.	Primary qualitative	Community members	Administrators of high schools, high school teachers, parents of high school students, and high school students.	84	FGDs and Individual interviews	Urban	Grounded theory	Minor
Woodland, 2022^55^ England (HIC)	Community	To investigate perceptions and experiences relating to the use of National Health Service Test & Trace (NHSTT) among parents of school-aged children (4 to 18 years) primarily to understand factors associated with COVID-19 symptom identification and the reasons why parents do or do not request a test when their child is symptomatic.	Primary qualitative	Community members	Parents of school-aged children (4–18 years)	18	Interview	Urban	Thematic analysis	Minor
Singh, 2021^56^ Nepal (LMIC)	Community	To explore community perceptions of COVID-19 and their experiences towards health services utilisation during the pandemic in Province-2 of Nepal.	Primary qualitative	Community members	Healthcare workers, female community health volunteers, local community representatives, teachers, social workers, and journalists.	14	Interview	Urban	Thematic analysis	Minor
Dodd, 2022^57^ The Philippines (LMIC)	Community	To expand understanding of who is actually involved in community health engagement efforts and the challenges they encounter in this work – an inquiry with implications for organisations charged with protecting the mental and physical well-being of their workers.	Primary qualitative	Community members	Community-based health actors.	28	Interview	Urban	Thematic analysis	Minor
Besselaar, 2022^58^ Netherlands (HIC)	Care homes	To evaluate how a national policy of testing for severe acute respiratory syndrome coronavirus 2 (SARS-CoV-2) regardless of symptoms was implemented during outbreaks in Dutch nursing homes in the second wave of the pandemic and to explore barriers and facilitators to serial testing.	Mixed method	Health workers	Direct care staff and management of care homes including: elderly care physicians, nurses, certified health assistants, board member, administrators.	52	Interview and FGDs	Urban and Rural	Thematic analysis	Minor
Blake, 2022^59^ England (HIC)	Community	To increase the frequency of asymptomatic SARS-CoV-2 saliva testing onsite.	Mixed method	Community member	Students and staff in a large England university campus.	43	Interview and FGDs	Urban	Thematic analysis	Minor
Mowbray, 2021^60^ England (HIC)	Community	To explore the key issues that underlie peoples’ engagement with National Health Service Test & Trace (NHSTT), specifically with regards to how people understand the symptoms that may indicate the presence of COVID-19 and that should trigger a request for a test.	Primary qualitative	Community member	General population and students.	40	Interview	Urban	Thematic analysis	Minor
Kas-Osoka, 2022^61^ US (HIC)	Community	To explore African Americans’ views towards COVID-19 testing and contact tracing to inform messaging and intervention targets.	Primary qualitative	Community member	African Americans.	62	Interview	Rural	Cross case analysis	Minor
Martindale, 2021^62^ England (HIC)	Healthcare facility	To assist with ongoing learning and to inform future pandemic diagnostic preparedness.	Primary qualitative	Health workers	Nurses, a dietician, a speech and language therapist, physicians, clinical directors and a GP partner.	13	Interview	Urban	Content analysis	Minor
Tulloch, 2021^63^ England (HIC)	Care homes	To evaluate outcomes in terms of preventing outbreaks, and process through the adoption of and adherence to the LFD testing regimens. We sought to understand behavioural, usability, administrative and organisational factors that might affect the testing process and its impact on COVID-19 prevention.	Mixed method	Health workers	Care homes staff including: managers, senior carer, staff nurses, and an administrator.	15	Interview	Urban	Thematic analysis	Minor

Note: Please see the full reference list of the article Nwachuku NS, Arikpo DI, Agbor UJ, et al. Factors influencing uptake of diagnostic test interventions for SARS-CoV-2: A qualitative review. J Public Health Africa. 2025;16(2), a619. https://doi.org/10.4102/jphia.v16i2.619, for more information.

LMIC, low- and middle-income countries; COVID-19, coronavirus disease 2019; SARS-CoV-2, severe acute respiratory syndrome coronavirus 2; HIC, high-income countries; US, United States; FGDs, focus group discussions; SDOH, social determinants of health; LFT, lateral flow testing; ST, self testing; GP, general practitioner; MAXQDA, Max Weber Qualitative Data Analysis; LCC, Liverpool City Council; LFD, lateral flow device.

All the 22 studies included for qualitative synthesis were primary qualitative studies. One of the studies collected local narratives from local community media and social media, while another study used an open-ended questionnaire format to collect qualitative data. The remaining 20 studies used focus group discussions (FGDs) and/or interviews to collect data (Table 2).

Among the sampled studies, two were conducted in healthcare facilities, two in care homes and 18 in community settings. Of the two health facility-based studies, the participants in one were recipients of care and the second were healthcare workers. The two care home studies involved different cadres of health workers directly and indirectly involved in patient care. Both studies focussed on implementation of testing policies. Participants in the community-based studies included the homeless and unsheltered (Table 2). Nineteen studies out of the 22 included reported on barriers and facilitators to uptake of diagnostic test interventions while 11 studies from the 22 included studies reported on perceptions and experiences of health workers, recipients of care and community members.

### Assessment of methodological quality

The methodological quality of the studies ranged from moderate limitations (*n* = 2) to no limitations (*n* = 2) and minor methodological limitations (*n* = 18) (Table 4). In the two studies with moderate methodological limitations and one study with minor methodological limitation, participants received monetary compensation for participation. Most studies provided descriptive information on the study context, sampling strategy, data collection and analysis approaches, and ethical considerations. They also offered basic data to support their findings. None of the studies clearly reported on researcher reflexivity. Table 2 details the characteristics of included studies and the overall assessment of methodological limitation.

### Qualitative synthesis findings

We identified 31 descriptive themes and refined these into 13 analytical themes (Table 3). The descriptive themes summarise perceptions, experiences and factors influencing uptake of diagnostic tests; the analytical themes represent re-grouped and refined descriptive themes. Using the GRADE CERQual tool, we assessed the confidence in each finding and graded 12 out of 13 findings as moderate confidence and one finding as low confidence. Table 4 presents a summary of the qualitative findings and CERQual assessments, and next we report the findings under each analytical theme.

**TABLE 3:** Synthesis results (themes and supporting quotes).

S. No.	Analytical themes	Descriptive themes (review findings)	Studies contributing to the review finding	Supporting data (example quote)
1	Testing provokes multiple fears among the public	Fear of contracting COVID-19 at testing centres	Garcini, 2022^23^ (US); Gehlbach, 2022^26^ (US); Knight, 2022^46^ (US); Woodland, 2022^55^ (England); Robin, 2022^50^ (England); Tonkin, 2022^53^ (Australia);	‘I would probably prefer to do a home test because, like I say, I think for me it would be a risk if I was to visit a test centre because there would be other people there who potentially have other symptoms and I could be exposed to them if I don’t have Covid, and then … Yes, I’m just putting myself in a vulnerable position, I think if I go to a test centre.’ (Woodland, 2022, England).
		Fear of the test procedure	Garcini, 2022^23^ (US); Gierszewski, 2022^45^ (Germany); DeRoo, 2021^51^ (US); Unger, 2021^54^ (US)	‘I heard that it is dangerous to have the test go down so far into your nose and it may choke you … they say they hurt you, that they make you bleed … I am afraid of doing the test myself.’ (Garcini, 2022, US)
		Fear of socio-economic implications of a positive test	Gehlbach, 2022^26^ (US); Garcini, 2022^23^ (US); Knight, 2022^46^ (US); Tonkin, 2022^53^ (Australia)	‘There is fear that you can lose your job if you test positive for the virus.’ (Gehlbach, 2022, US).
		Fear of racism	DeRoo, 2021^51^ (US)	‘When you have a Black patient and you have a White doctor, you have the Black patient saying they have all these symptoms, and the White doctor isn’t taking it very seriously and whatnot. Why should I even worry about getting this test or whatnot?’ (DeRoo, 2021 US).
		Fear of immigration status	Gehlbach, 2022^26^ (US)	‘Many people who live in [*X community*], they have no ID. So many people don’t get the test because they don’t have documents, and they don’t have identification.’ (Gehlbach, 2022, US)
2	Beliefs and behaviour surrounding testing	Perceived threat and susceptibility to COVID-19	Knight,^52^ 2022^46^ (US); Lorenc 2021^47^ (England); Nwaozuru 2022^49^ (US); DeRoo, 2021^51^ (US); Woodland 2022^55^ (England); Tonkin 2022^53^ (Australia)	‘I actually am high risk too because I have co-morbidities. So, if I catch [*COVID*] I’m at risk for severe illness … Diabetes, high blood pressure and cholesterol. The trifecta.’ (Knight, 2022, US)
		Avoiding implications of a positive result	Mathers 2022^48^ (England); Thorneloe 2022 (England); Unger 2021^54^ (US); Woodland 2022^55^ (England); Dodd 2022^57^ (The Philippines)	Actually, I was also one of those hesitant to have the swab test because [*if*] you test positive, everyone will be affected … So, if I tested positive, all of the people in our compound will be affected. All of us will be quarantined. That was my worry. That was also the feeling of other staff, if they test positive […] …, we are very crowded (Dodd 2022, The Philippines).
		Testing to protect others and self	Robin 2022^50^ (England); Garcini 2022^23^ (US); Tonkin 2022^53^ (Australia); Tulloch, 2021^63^ (England)	… it is also my obligation to the South Australian government, to the Australian Community, that I get tested and that I’m not a risk to not only the people around me but the people that I come into contact with … So, it’s also a moral obligation to the place I live to … to make sure that I follow the guidelines, um … Because they’re not pushing us to get tested, they’re asking us to get tested, so, um, it’s my moral obligation, um, for the community, the wider community, that I make sure I’m not a risk to them, too (Tonkin, 2022, Australia).
		Asymptomatic testing makes one feel safe	Mathers 2022^48^ (US); Mowbray, 2021^60^ (England); DeRoo, 2021^51^ (US); Thorneloe, 2022^52^ (England)	Gives me peace of mind that I’m not going to spread it without symptoms … that other people in the office are testing and I can safely interact with them, and I know that if I go to the supermarket or see someone not in my household I know I’m not going to spread it to them as well. (ID 8, testing) (Matthers 2022; US).
3	Testing preferences	Preference for less invasive tests	Blake 2022^59^ (England); Unger 2022^54^ (US); Nwaozuru 2022^49^ (US)	‘The saliva test was really, it’s really easy to do and it’s not like uncomfortable like the swab tests so, yeah, I much prefer doing them.’ (Blake, 2022, England).
		Discomfort from test procedure	Mathers 2022^48^ (US); Van de Besselaar 2012^58^ (the Netherlands); Tonkin 2022^53^ (Australia)	Still makes me heave and eyes water, but the feeling passes quickly, and a small price to pay if COVID-19 infections are prevented by the testing strategy (Matthers, 2022, US).
4	Questioning the need for testing	No need for asymptomatic testing	Blake, 2022^59^ (England); Nwaozuru, 2022^49^ (US); Knight, 2022^46^ (US); Mathers, 2022^48^ (England); DeRoo, 2021^51^ (US)	‘I’m not going out so not something that I’ve needed to have … if I haven’t got symptoms and I’m not going anywhere, why do I need a test?’ (Mathers, 2022, England)
		Vaccination removes the need for testing	Mathers, 2022^48^ (England); Tonkin, 2022^53^ (Australia)	‘I’d expect that the vaccine wouldn’t let me get COVID. I would expect that’s its whole purpose, so I wouldn’t-test if I was vaccinated’ (Tonkin, 2022, Australia).
		Testing does not stop transmission	Mathers, 2022^48^ (England); Robin, 2022^50^ (England)	… I think people actually believe that the testing is a way to stop transmission, and I’m not totally convinced … I feel that LFT may have a role in reducing transmission, but that comes at a cost and I feel it’s not OK to discuss that cost. (Mathers, 2022, England).
5	Deciding whether to test	Testing based on self-assessment of symptoms	Mowbray, 2021^60^ (England); Nwaozuru, 2022^49^ (US); DeRoo, 2021^51^ (US) Thorneloe, 2022^52^ (England); Tonkin, 2022^53^ (Australia); Woodland 2022^55^ (England)	‘I would only do it if the temperature was high and I had a continuous cough as well and I’d been out with my friends. If I had the symptoms then I would go and get tested, just to make sure that I was safe.’ (Participant 146, Student Mowbray, 2021, England).
6	In principle support for diagnostic testing	Testing is useful, and support for frequent or universal testing	Mathers, 2022^48^ (England); Gierszewski, 2022^45^ (Germany); Unger, 2021^54^ (US); Lorenc, 2021^47^ (England); Robin, 2022^50^ (England); Knight, 2022^46^ (US)	‘I wouldn’t mind testing every day. I think it gives you more reassurance that you’re not positive. Testing every day, for me, won’t be a problem. I would feel more secure, and if I have to be in the classroom, if students are tested every day, I would feel more comfortable. Otherwise, you never know when they get it. So every day is probably more secure.’ (Unger, 2021, US).
7	Concerns about test accuracy and reliability	Concern about test accuracy and reliability of results	Garcini 2022^23^ (US); Gierszewski 2022^45^ (Germany); Knight 2022^46^ (US); Mathers 2022^48^ (England); DeRoo, 2021^51^ (US); Thorneloe 2022^52^ (England); Van de Besselaar 2021^58^ (the Netherlands); Robin 2022^50^ (England); Tonkin 2022^53^ (Australia)	‘… The tests have very high false positives and they’ve even got false negatives as well. So you can’t, you wouldn’t be able to rely on the test anyway ….’ (Thorneloe, 2022, England)
		LFTs less accurate than PCRs	Mathers, 2022^48^ (England)	I know that the LFTs aren’t 100% but if it identifies one person whose got it who if they didn’t know about it could’ve spread it then it’s worthwhile isn’t it.’ (Mathers, 2022, England).
		Lack of confidence in self-testing at home	Garcini, 2022^23^ (US); Mowbray, 2021^60^ (England); Nwaozuru, 2022^49^ (US); DeRoo, 2021^51^ (US); Woodland, 2022^55^ (England)	‘Not 100 percent accurate, potentially people won’t perform the test correctly’ (19 years, Female, Maryland)*Could mess up the sample, not enough knowledge on self-testing, so it could potentially be a problem*.’ (Nwaozuru, 2022, US).
8	Convenience of testing	Logistics and convenience of testing centres	Bateman, 2021^44^, (US); Garcini, 2022^23^, (US); Thorneloe, 2022^52^, (England); Woodland, 2022^55^, (England); Robin, 2022^50^, (England); Unger, 2021^54^, (US); Tulloch, 2021^63^ (England)	‘There’s concern about the testing sites being accessible still in all communities … If you are a senior, and you don’t have your own car, and you aren’t able to take a bus to that location … the logistics of the testing situation are just not amenable.’ (Bateman 2021, US)
		Positive experience of testing	Blake, 2022^59^, (England); Mathers, 2022^48^, (England); Robin, 2022^50^, (England)	‘Once I had the test it took under an hour for the result to come through … so this test could be a game changer ….’ (Robin, 2022, England)
9	Opportunity costs	Opportunity cost of self-isolating	Tonkin, 2022^53^, (Australia)	‘I had an exam in between and I couldn’t attend it because I had to be home up until my results were out.’ (Tonkin, 2022, Australia)
10	Affordability	Rumours about the cost of self-testing	Garcini, 2022^23^ (US); Nwaozuru, 2022^49^ (US); Singh, 2021^56^ (Nepal); Unger, 2021^54^ (US); Bateman, 2021^44^ (US)	‘The testing itself is expensive. And I’m not quite sure if it’s sustainable. Of course, if it’s free, then you provide it. But I don’t have funds for $5 per student … But if they’re asking the school or the district to pay for it, there is a pretty substantial cost. We can figure out logistics. It’s how to pay for it. That becomes a challenge for me.’ (Unger, 2021, US).
11	Service delivery factors influencing uptake of testing	Queues and waiting times at test centres	Garcini 2022^23^ (US); Thorneloe 2022^52^ (England); Robin 2022^50^ (England); Tonkin 2022^53^ (Australia); Tulloch, 2021^63^ (England)	‘Like as much as I’d want to do my part in it, is it worth going to all the trouble because it’s long queues to get a test. You might have to go to [*a different town*] for a test, like, it’s not a local kind of thing most of the time, sometimes.’ (Thorneloe, 2022, England)
		Availability and accessibility of testing	Garcini 2022^23^ (US); Bateman 2021^44^ (US); Robin 2022^50^ (England); Van de Besselaar 2021^58^ (the Netherlands)	‘There are not enough tests … many times [*people*] do not get tested because by the time their turn comes, they are turned away and told there are no more tests available, even though they are getting up very early to get tested.’ (Garcini, 2022, US)
		Long wait times for test results	Martindale 2021^62^ (England); Singh 2021^56^ (Nepal); Woodland 2022^55^ (England); Thorneloe 2022^52^ (England)	‘I’m not sure. If anything, it makes me want to definitely double check that they do have a temperature before I even suggest that we get tested because we have to wait for the test results. I couldn’t go into work for two days until I got my test results back when I had my test, so that was not particularly great … It meant that I couldn’t drop my kids off at school. My husband had to leave for work late because he had to do that because I wasn’t allowed to leave the house. Yes, I definitely would double check that there’s definitely something wrong with them before I think about making it official and getting tested and telling playgroup or nursery.’ (Woodland, 2022, England)
12	Policy and political factors	Lag between policy and implementation	Martindale 2021^62^ (England);	‘[*I*]t isn’t about the advice, it is all about the implementation and implementation is difficult, reaching out to every MP, every hospital, every manufacturer is not easy … but there has been too much of a separation of advice, lag phase, implementation and we can’t get that wrong … otherwise we will go very quickly back into a rebound.’ (Martindale, 2021, England)
		Mandates and incentives for testing	DeRoo, 2021^51^ (US); Singh 2021^56^ (Nepal); Robin 2022^50^ (England); Bateman 2021^44^ (US); Knight 2022^46^ (US)	‘ … I guarantee you, if you give some type of incentive, they will get there and test’ (female, age 50), and ‘ … the truth is, we’ve had a legacy of people coming into our communities sprinkling trinkets, and our folks have gotten used to it.’ (Bateman, 2021, US)
		Lack of trust in government and health workers	Bateman 2021^44^ (US); Robin 2022^50^ (England)	‘To be very honest—and it’s getting back to a whole lot of things that have happened to our people back in the day. They don’t trust doctors. They don’t trust people … I haven’t taken the test. I don’t know if I’ll take the test.’ (Bateman, 2021, US)
13	Social factors	Misconceptions and rumours	Garcini, 2022^23^, (US); Knight, 2022^46^, (US); DeRoo, 2021^51^, (US); Gehlbach, 2022^26^, (US); Bateman, 2021^44^, (US); Robin, 2022^50^, (England)	‘[*People*] think they’re getting tested and they could be given the virus … Because they have ways of giving people this virus and they don’t know how they’re getting it, but that’s one way they can do that.’ (Knight, 2022, US)
		Stigma and discrimination	Dodd, 2022^57^ (The Philippines); Garcini 2022^23^ (US); Mowbray 2021^60^ (England); DeRoo, 2021^51^ US; Unger^54^ 2021 (US); Woodland 2022^55^ (England); Mathers^48^ 2022 (US)	‘The discrimination was the hardest deal for me […]. [*Community members*] were saying if they saw me go out of the house they would chop my legs off. They were saying I am useless; why did I go out? So I defended myself saying that the time when I went to an area or left the house, I was not positive at that time. […] The sad part was that they knew you were down, but they didn’t care, and no one ever wanted to be positive, not even me (PS025).’ (Dodd 2022, The Philippines)

Note: Please see the full reference list of the article Nwachuku NS, Arikpo DI, Agbor UJ, et al. Factors influencing uptake of diagnostic test interventions for SARS-CoV-2: A qualitative review. J Public Health Africa. 2025;16(2), a619. https://doi.org/10.4102/jphia.v16i2.619, for more information.

S. No., serial number; LFTs, lateral flow testing; PCRs, polymerase chain reaction; US, United States.

**TABLE 4:** Summary of qualitative findings and CERQual assessment.

S. No.	Analytical themes	Studies contributing to the review finding	Supporting data (example quote)	GRADE CERQual assessment of confidence in the evidence	Explanation of the CERQUAL assessment
1	Testing provokes multiple fears among the public	DeRoo, 2021^51^ (US); Garcini, 2022^23^ (USA); Gehlbach, 2022^26^ (US); Gierszewski, 202245 (Germany); Knight, 2022^46^ (US); Robin, 2022^50^ (England); Tonkin, 2022^53^ (Australia); Unger, 2021^54^ (US); Woodland, 2022^55^ (England)	‘I heard that it is dangerous to have the test go down so far into your nose and it may choke you … they say they hurt you, that they make you bleed … I am afraid of doing the test myself.’ (Garcini, 2022, US)	Moderate confidence	No to very minor concerns regarding coherence and adequacy, minor concerns regarding methodological limitations and moderate concerns regarding relevance (limited geographical spread)
2	Beliefs and behaviour surrounding testing	DeRoo, 2021^51^ (US); Dodd 2022^57^ (The Philippines); Garcini 2022^23^ (US); Knight 2022^46^ (US); Lorenc 2021^47^ (England); Mathers 2022^48^ (England); Mowbray, 2021^60^ (England); Nwaozuru 2022^49^(USA); Robin, 2022^50^ (England); Tonkin 2022^53^ (Australia); Thorneloe, 2022^52^ (England); Tulloch, 2021^63^ (England); Unger 2021^54^ (US); Woodland 2022^55^ (England)	Actually, I was also one of those hesitant to have the swab test because [if] you test positive, everyone will be affected … So, if I tested positive, all of the people in our compound will be affected. All of us will be quarantined. That was my worry. That was also the feeling of other staff, if they test positive […] …, we are very crowded.’ (Dodd 2022, The Philippines)	Moderate confidence	No to very minor concerns regarding coherence and adequacy, minor concerns regarding methodological limitations and moderate concerns regarding relevance (limited geographical spread)
3	Testing preferences	Blake 2022^59^ (England); Mathers, 2022^48^ (US); Nwaozuru 2022^19^ (USA); Tonkin 2022^53^ (Australia); Unger 2022^54^ (US); Van de Besselaar 2012^58^ (the Netherlands)	‘The saliva test was really, it’s really easy to do and it’s not like uncomfortable like the swab tests so, yeah, I much prefer doing them.’ (Blake, 2022, England)	Moderate confidence	No to very minor concerns regarding coherence and adequacy, minor concerns regarding methodological limitations and moderate concerns regarding relevance (limited geographical spread)
4	Questioning the need for testing	Blake, 2022^59^ (England); DeRoo, 2021^51^ (US); Knight, 2022^46^ (US); Mathers, 2022^48^ (England); Nwaozuru, 2022^49^ (USA); Robin, 2022^50^ (England); Tonkin, 2022^53^ (Australia)	‘I’m not going out so not something that I’ve needed to have … if I haven’t got symptoms and I’m not going anywhere, why do I need a test?’ (Mathers, 2022, England)	Moderate confidence	No to very minor concerns regarding coherence and adequacy, minor concerns regarding methodological limitations and moderate concerns regarding relevance (limited geographical spread)
5	Deciding whether to test	Mowbray, 2021^60^ (England); Nwaozuru, 2022^49^ (US); DeRoo, 2021^51^ (US) Thorneloe, 2022^52^ (England); Tonkin, 2022^53^ (Australia); Woodland 2022^55^ (England)	‘I would only do it if the temperature was high and I had a continuous cough as well and I’d been out with my friends. If I had the symptoms then I would go and get tested, just to make sure that I was safe.’ (Participant 146, Student, Mowbray, 2021, England)	Moderate confidence	No to very minor concerns regarding coherence and adequacy, minor concerns regarding methodological limitations and moderate concerns regarding relevance (limited geographical spread)
6	In principle support for diagnostic testing	Mathers, 2022^48^ (England); Gierszewski, 2022^45^ (Germany); Unger, 2021^54^ (US); Lorenc, 2021^47^ (England); Robin, 2022^50^ (England); Knight, 2022^46^ (US)	‘I wouldn’t mind testing every day. I think it gives you more reassurance that you’re not positive. Testing every day, for me, won’t be a problem. I would feel more secure, and if I have to be in the classroom, if students are tested every day, I would feel more comfortable. Otherwise, you never know when they get it. So every day is probably more secure.’ (Unger, 2021, US)	Moderate confidence	No to very minor concerns regarding coherence and adequacy, minor concerns regarding methodological limitations and moderate concerns regarding relevance (limited geographical spread)
7	Concerns about test accuracy and reliability	DeRoo, 2021^51^ (US); Garcini 2022^23^ (US); Gierszewski 2022^45^ (Germany); Knight 2022 (US); Mathers 2022^48^ (England); Mowbray, 2021^60^ (England); Nwaozuru, 2022^49^ (US); Robin 2022^50^ (England); Thorneloe 2022^52^ (England); Tonkin 2022^53^ (Australia); Van de Besselaar 2021^58^ (the Netherlands); Woodland, 2022^55^ (England)	‘…The tests have very high false positives and they’ve even got false negatives as well. So you can’t, you wouldn’t be able to rely on the test anyway …’ (Thorneloe, 2022, England)	Moderate confidence	No to very minor concerns regarding coherence and adequacy, minor concerns regarding methodological limitations and moderate concerns regarding relevance (limited geographical spread)
8	Convenience of testing	Bateman, 2021^44^, (US); Blake, 2022^59^, (England); Garcini, 2022^23^, (US); Mathers, 2022^48^, (England); Robin, 2022^50^, (England); Thorneloe, 2022^52^, (England); Tulloch, 2021^63^ (England); Unger, 2021^54^, (US); Woodland, 2022^55^, (England);	There’s concern about the testing sites being accessible still in all communities … If you are a senior, and you don’t have your own car, and you aren’t able to take a bus to that location … the logistics of the testing situation are just.’ (Bateman 2021, US)	Moderate confidence	No to very minor concerns regarding coherence and adequacy, minor concerns regarding methodological limitations and moderate concerns regarding relevance (limited geographical spread)
9	Opportunity costs	Tonkin, 2022^53^, (Australia)	‘I had an exam in between and I couldn’t attend it because I had to be home up until my results were out.’ (Tonkin, 2022, Australia)	Low confidence	No to very minor concerns regarding coherence, minor concerns regarding methodological limitations. Moderate concerns regarding relevance (limited geographical spread) and adequacy
10	Affordability	Bateman, 2021^44^ (US); Garcini, 2022^23^ (US); Nwaozuru, 2022^49^ (US); Singh, 2021^56^ (Nepal); Unger, 2021^54^ (US)	‘The testing itself is expensive. And I’m not quite sure if it’s sustainable. Of course, if it’s free, then you provide it. But I don’t have funds for $5 per student … But if they’re asking the school or the district to pay for it, there is a pretty substantial cost. We can figure out logistics. It’s how to pay for it. That becomes a challenge for me.’ (Unger, 2021, US)	Moderate confidence	No to very minor concerns regarding coherence and adequacy, minor concerns regarding methodological limitations and moderate concerns regarding relevance (limited geographical spread)
11	Service delivery factors influencing uptake of testing	Bateman 2021^44^ (US); Garcini 2022^23^ (US); Martindale 2021^62^ (England); Robin 2022^50^ (England); Singh 2021^56^ (Nepal); Thorneloe 2022^52^ (England); Tonkin 2022^53^ (Australia); Tulloch, 2021^63^ (England); Van de Besselaar 2021^58^ (the Netherlands); Woodland 2022^55^ (England)	‘Like as much as I’d want to do my part in it, is it worth going to all the trouble because it’s long queues to get a test. You might have to go to [*a different town*] for a test, like, it’s not a local kind of thing most of the time, sometimes.’ (Thorneloe, 2022, England)	Moderate confidence	No to very minor concerns regarding coherence and adequacy, minor concerns regarding methodological limitations and moderate concerns regarding relevance (limited geographical spread)
12	Policy and political factors	Bateman 2021^44^ (US); DeRoo, 2021^51^ (US); Knight 2022^46^ (US); Martindale 2021^62^ (England); Robin 2022^50^ (England); Singh 2021^56^ (Nepal)	‘[*I*]t isn’t about the advice, it is all about the implementation and implementation is difficult, reaching out to every MP, every hospital, every manufacturer is not easy … but there has been too much of a separation of advice, lag phase, implementation and we can’t get that wrong … otherwise we will go very quickly back into a rebound.’ (Martindale, 2021, England)	Moderate confidence	No to very minor concerns regarding coherence and adequacy, minor concerns regarding methodological limitations and moderate concerns regarding relevance (limited geographical spread)
13	Social factors	Bateman, 2021^44^, (US); DeRoo, 2021^51^, (US); Dodd, 2022^57^, (The Philippines); Garcini, 2022,^23^ (US); Gehlbach, 2022^26^, (US); Knight, 2022^46^, (US); Mathers 2022^48^ (US); Mowbray 2021^60^ (England); Robin, 2022^50^, (England); Unger 2021^54^ (US); Woodland 2022^55^ (England)	The discrimination was the hardest deal for me […]. [*Community members*] were saying if they saw me go out of the house they would chop my legs off. They were saying I am useless; why did I go out? So I defended myself saying that the time when I went to an area or left the house, I was not positive at that time. […] The sad part was that they knew you were down, but they didn’t care, and no one ever wanted to be positive, not even me.’ (PS025) (Dodd 2022, The Philippines)	Moderate confidence	No to very minor concerns regarding coherence and adequacy, minor concerns regarding methodological limitations and moderate concerns regarding relevance (limited geographical spread)

Note: Please see the full reference list of the article Nwachuku NS, Arikpo DI, Agbor UJ, et al. Factors influencing uptake of diagnostic test interventions for SARS-CoV-2: A qualitative review. J Public Health Africa. 2025;16(2), a619. https://doi.org/10.4102/jphia.v16i2.619, for more information.

S. No., serial number; COVID-19, coronavirus disease 2019; US, United States; LFTs, lateral flow testing; PCRs, polymerase chain reaction; GRADE, Grading of Recommendations Assessment, Development, and Evaluation; CERQUAL, Confidence in the Evidence from Reviews of Qualitative Research.

#### Finding 1: Testing provokes multiple fears among the public (moderate confidence)

In nine studies conducted in HICs, community members expressed fear of the risk of contracting COVID-19 at test centres, preferring a home test instead. In addition, reservations were expressed about the test procedure because of discomfort and pain. Another form of fear expressed was the socio-economic implications of having a positive test result. Individuals worried they could lose their income or job because of their inability to work. Interestingly, these multiple fears were expressed across all the studies in HICs.

In two studies conducted in US among Latinx and Indigenous Latin American immigrant communities and black adults, individuals were hesitant to get tested because of their immigration status. They feared that turning up for a test would put them at risk of being identified by the authorities because they were unsure if testing service would share their personal data. Perceived systemic racism made them less prioritised for COVID-19 care.

#### Finding 2: Beliefs and behaviour surrounding testing (moderate confidence)

Evidence from 14 studies conducted in 3 HICs and 1 LMIC showed that the perceived threat of and susceptibility to COVID-19 motivated community members to get tested. Persons with co-morbidity felt vulnerable and at high risk of severe illness if they got infected with the virus. Some persons were unwilling to test in a bid to avoid the implications of a positive test result on their immediate community. They feared they would be quarantined if anyone tested positive because they lived or worked in crowded environments. However, in contrast to the previous perception, some community members felt the moral obligation to get tested to protect their family, the vulnerable and their immediate community. For others, a negative test result was reassuring because they will not be a source of infection in the household, community or workplace. These perceptions reflect responses from studies from both HICs and LMICs.

#### Finding 3: Testing preferences (moderate confidence)

Evidence from six studies conducted in HICs revealed the preference for less invasive tests such as the saliva test compared to the swab test by community members and residents of care homes. Saliva tests were also perceived to be more convenient; although, participants generally complained of discomfort such as pain, which are transient.

#### Finding 4: Questioning the need for testing (moderate confidence)

Seven studies reporting community members’ perspectives conducted from HICs questioned the need for asymptomatic testing when they had followed the COVID-19 guidelines, were never sick, never had any symptoms or knew any confirmed cases of COVID-19. There was also a perception that vaccination removed the need for testing hence the question on why vaccinated persons should get tested, because testing does not stop transmission or remove the likelihood of getting re-infected with the virus.

#### Finding 5: Deciding whether to test (moderate confidence)

Six studies conducted in HICs show the decision to get tested was largely informed by the individual’s self-assessment of symptoms. Willingness to get tested was based on having two or more classic symptoms of COVID-19 or felt sick enough, especially after possible exposure.

#### Finding 6: In principle support for diagnostic testing (moderate confidence)

Community members in six studies, conducted in HICs, expressed support for frequent or universal asymptomatic testing. This is because they perceived frequent testing (daily or weekly) would give them a sense of reassurance that they were not infected especially if they had interacted with others socially.

#### Finding 7: Concerns about test accuracy and reliability (moderate confidence)

Eleven studies conducted in HICs expressed concerns about the accuracy and reliability of test results from community members and healthcare workers. These concerns include high rates of false positives and false negative results. In addition to this was the cost of having to repeat the test when the result is unreliable or decisions are made based on inaccurate results. Community members also perceived lateral flow tests to be less accurate than polymerase chain reaction (PCR) test and were not confident in their ability to conduct self-tests at home.

#### Finding 8: Convenience of testing (moderate confidence)

Nine studies from HICs, consisting of one study involving staff of care homes and eight involving community members, reported concerns about accessibility; particularly vehicular access and convenience of testing centres in the community especially for the elderly. The study involving care home staff from England was concerned about inconvenience of managing testing procedures.

#### Finding 9: Opportunity costs (low confidence)

One study conducted in Australia showed there was an opportunity cost associated to testing which was the cost of isolating while waiting for test results. Community members complained about the inability to sit for an examination as it was necessary to self-isolate until the test result is out, and the increased cost of living because meals had to be ordered from food outlets.

#### Finding 10: Affordability (moderate confidence)

Five studies, four from a HIC and one from LMIC, among community members were concerned about the perceived high cost of self-testing and its sustainability. In their opinion, free tests will motivate more persons to get tested.

#### Finding 11: Service delivery factors influencing uptake of testing (moderate confidence)

Ten studies from four HICs and one in a LMIC reported several service delivery factors including availability, accessibility, queues and long waiting time for tests and test results.

Although they desired to be tested, their experience of long queues after which they may still not get tested discouraged testing. Sometimes getting tested required commuting to another town. These barriers coupled with the anticipated consequences of a positive test (isolation), conditioned people to only seek tests based on symptom recognition.

#### Finding 12: Policy and political factors (moderate confidence)

Evidence from six studies illustrates the gap between policy and implementation, the influence of incentives, as well as the lack of trust in government and health workers as socio-political factors influencing diagnostic test uptake. These studies were conducted in two HICs and one LMIC. Two studies, one from an HIC and another from an LMIC, show that government mandate can either discourage or prompt people to test. In addition, the study from LMIC strongly expressed a lack of synergy among the tiers of government regarding testing. A study from an HIC expressed strong reservation about forceful testing mandate and another pointed out corruption in government and the lack of trust for health workers (Doctors) and people based on the Tuskegee incident where people of colour were used as experimental beings rather than humans for testing as barriers.

#### Finding 13: Social factors (moderate confidence)

Eleven studies, conducted in two HICs and one in an LMIC, identified misconceptions, conspiracy theories, rumours, stigmatisation and discrimination of persons who got tested or persons who tested positive. Therefore, community members avoided getting tested for fear of being shamed, blamed, isolated and stigmatised. Individuals also experienced backlash and blame from health workers for not following guidelines and putting others at risk in the US. Because of this anticipated fear, those who had tested, concealed from others that they had been tested, while others out of fear opted not to get tested.

## Discussion

### Summary of main results

Summary of main results are outlined in Table 4.

### Description of studies

Our review aimed to identify and synthesise the findings of qualitative studies on the perceptions, experiences and views of community members, healthcare providers and recipients of care on diagnostic test interventions, and on barriers and facilitators to uptake of diagnostic test interventions in the context of COVID-19. Most of the studies were conducted in HICs (US, England, and Europe) and explored views and perspectives of community members in the community setting.

### Factors influencing uptake

Major themes of low to moderate quality evidence centred around fear of the implication of a positive test, beliefs, misconceptions and myths, concern about test accuracy and reliability, convenience and cost of testing, policy and implementation issues, as well as social, political and community factors.^19,64,65^ The implication of this finding, hinges on the role of fear and misinformation as barriers to the uptake of diagnostic tests among the sampled population. The consequences of a positive test (quarantine) which isolates the individual from family and community members were not acceptable. The accuracy and reliability of the antibody and antigen tests were questioned probably because of the variation in test results on account of the antibody level in the antibody tests and viral load in the antigen tests. Other barriers to uptake were fear of testing procedures, and discomfort associated with testing, which were considered too invasive and uncomfortable especially for the nasopharyngeal or oropharyngeal swabs.^66,67^ Some questioned the need for asymptomatic testing.^68,69^ However, others expressed support for frequent or universal asymptomatic testing because it gave them a sense of reassurance.^70,71^ This review also identified conspiracy theories, illegal immigration status and perceived racism as barriers.

Overall, fear and beliefs are prominent in high-income settings especially fear and belief of perceived vulnerability and susceptibility. In HICs where we found information, people questioned the need for testing when other control measures are in place (e.g. vaccines). Questioning the need for testing may be linked to misconceptions largely centred on symptom presentation and the lack of clarity of government policy in some countries on testing for symptomatic and asymptomatic individuals. Concerns about accuracy and reliability are real and are attributed to the high number of false positive and negative tests. Service delivery factors expressed as barriers were mainly availability and accessibility.

This review has consolidated findings on community-level misconceptions, conspiracy theories and rumours that appear to extend across HICs and LMICs. Despite wide spread fears and concern about diagnostic test for COVID-19 among community members, some respondents supported testing and saw the need to be tested to protect family members and the community and have reassurance that they are not positive for severe acute respiratory syndrome coronavirus-2 (SARS-CoV-2).

### Strengths of the study

The comprehensive search, we think identified almost all the qualitative studies conducted and reported in English during the period. We used the SPICE framework to help shape the review question and articulate the inclusion criteria.

### Limitations of the study

The rapid search method and our search of the English language literature only, meaning that we may have missed eligible studies and other studies reported in other languages – for example from Francophone West Africa, and Latin America. This review included studies mostly carried out in developed countries, and none from Africa. The two studies from LMICs, contributed very little to the review findings. Furthermore, there was insufficient evidence from health workers perspective.

### Gaps and implications for practice, policy and research

The implication of our review findings for practice, points to the need for educating communities and providing more information and health promotion material to counteract the misconceptions, rumours as well as beliefs and fears surrounding testing. For policy, findings suggests a gap between policy and implementation, the influence of incentives, as well as the lack of trust in government and health workers. Therefore, well defined polices with stakeholder engagement and a robust implementation monitoring strategy to get feedback can mitigate this gap. Although this finding is from HICs, it may be applicable to other settings. For research, there is the need for more studies in the healthcare setting, involving healthcare workers perspective as these are crucial stakeholders in IPC. The two studies from Nepal and The Philippines contributed to four themes, but we cannot be certain these reflect experiences and perceptions in other LMICs. Should another pandemic occur there is a need to deploy rapid qualitative methods quickly and in a coordinated way across multiple countries, especially in Africa. This should include ‘social listening’ and monitoring of social media for real-time on the ground beliefs and perceptions.

## Conclusion

Uptake of diagnostic test interventions were influenced by multiples factors, operating at the individual, community and institutional level.

Emerging themes revolved around fear of test procedure and socio-economic implication of positive test, beliefs of vulnerability and susceptibility, test preferences, accuracy, reliability, cost and affordability, testing experiences, service delivery factors such as long queues at testing centres, social, political and community factors, all shaped perception and uptake. These were largely based on misconception, misinformation, and the lack of trust from community members, while adaptation to managing testing procedures, implication of positive test to the health workforce and frequent changes to guideline were important factors that shaped support or otherwise for diagnostic test from health workers.

In conclusion, this review found a low to moderate quality evidence of barriers to uptake of diagnostic testing largely because of misconceptions about the intervention. It is recommended that community sensitisation targeting misconceptions and stakeholder engagement among healthcare workers will improve uptake and bridge the policy implementation gap. Applicability of our review findings in LMICs is limited because only two studies from LMICs contributed data to this QES.
